# Prevalence of type 2 diabetes from 2011 to 2023 by regional socioeconomic deprivation in Germany: an ecological study

**DOI:** 10.1186/s12889-025-25908-x

**Published:** 2025-12-17

**Authors:** Marielle Wirth, Saskia Bischoff, Ramona Hering, Mandy Schulz, Malwina Mackowiak, Katharina Piedboeuf-Potyka, Ralph Brinks, Annika Hoyer, Thaddäus Tönnies

**Affiliations:** 1 Institute for Biometrics and Epidemiology, German Diabetes Center (DDZ), Leibniz Center for Diabetes Research at Heinrich Heine University Düsseldorf, Düsseldorf, 40225 Germany; 2 Department of General Pediatrics, Neonatology and Pediatric Cardiology, Medical Faculty, University Hospital Düsseldorf, Heinrich Heine University Düsseldorf, Düsseldorf, 40225 Germany; 3 Medical Faculty, Heinrich Heine University Düsseldorf, Düsseldorf, 40225 Germany; 4 Department of Data Science and Healthcare Analyses, Central Research Institute for Ambulatory Health Care in Germany, Berlin, 10587 Germany; 5 Department of Mathematics, Informatics, Technology, Koblenz University of Applied Sciences, RheinAhrCampus, Remagen, 53424 Germany; 6 Chair for Medical Biometry and Epidemiology (MBE), Faculty of Health/School of Medicine, Witten/Herdecke University, Witten, 58448 Germany; 7 Biostatistics and Medical Biometry, Medical School OWL, Bielefeld University, Bielefeld, 33615 Germany

**Keywords:** Diabetes mellitus, Prevalence, Deprivation, Diabetes-surveillance

## Abstract

**Background:**

Type 2 diabetes prevalence is increasing globally and is unequally distributed across socioeconomic groups. In Germany, little is known about how these socioeconomic inequalities have developed over time. Therefore, we aimed to estimate the age- and sex-specific prevalence of type 2 diabetes in Germany from 2011 to 2023 and assess related temporal trends in socioeconomic inequality.

**Methods:**

We used nationwide claims data (*N* ~ 70 million persons per year) from the German statutory health insurance, aggregated at the county level and linked them to the German Index of Socioeconomic Deprivation (GISD), an area-based measure of deprivation based on education, occupation, and income. We identified prevalent type 2 diabetes cases using ICD-10 codes E11, E12, E13, and E14. Log-binomial regression was used to estimate prevalence ratios for the association between GISD quintiles and type 2 diabetes prevalence, with temporal trends assessed through the four-way interaction between age, sex, GISD, and calendar year.

**Results:**

Crude type 2 diabetes prevalence increased from 9.00% (95% confidence interval: 8.99–9.01) in 2011 to 9.60% (9.59–9.61) in 2023, while age-standardised prevalence showed a smaller increase, from 9.00 (8.99-9.00) in 2011 to 9.35% (9.34–9.35) in 2023. A clear socioeconomic gradient emerged, with the highest prevalence of 11.80% (11.78–11.82) in 2023 in the most deprived regions (quintile 5), compared to 8.00% (7.99–8.01) in the least deprived regions (quintile 1). Prevalence was consistently higher in men than women, but the socioeconomic gradient was more pronounced among women (prevalence ratio 2023 women 1.29 (1.23–1.35), men 1.22 (1.17–1.26)).

**Conclusions:**

Our findings highlight persistent socioeconomic disparities in type 2 diabetes prevalence, especially among women. Further research is needed to explore the mechanisms underlying these disparities and to evaluate targeted interventions for high-risk populations.

**Supplementary Information:**

The online version contains supplementary material available at 10.1186/s12889-025-25908-x.

## Introduction

The prevalence of type 2 diabetes has been increasing worldwide, posing a significant challenge to healthcare systems [1]. In Germany, the prevalence of type 2 diabetes was estimated at 9.5% in 2023 [2]. Projections suggest that the number of people with type 2 diabetes could increase between 10.9 million and 14.2 million by 2040, depending on trends in incidence and mortality [3]. Socioeconomic position (SEP) and regional deprivation – both indicators of social inequalities – are known to be associated with type 2 diabetes prevalence in Germany [4–6]. While SEP encompasses individual factors such as education, income and occupation, regional deprivation reflects area-level factors such as unemployment rates, household income or poverty rates [7]. To assess socioeconomic conditions across Germany, the German Index of Socioeconomic Deprivation (GISD) was developed [8]. By integrating indicators of income, employment status, educational attainment, and housing conditions, the GISD provides a comprehensive measure of socioeconomic disadvantage at the regional level. This index has proven to be a valuable tool in evaluating the impact of regional deprivation on health outcomes, including type 2 diabetes prevalence [9].

Despite the established association between regional deprivation and type 2 diabetes, little is known about how type 2 diabetes prevalence has evolved over time across regions with different levels of socioeconomic disadvantage in Germany. Examining these long-term trends provides valuable insights into whether socioeconomic disparities in type 2 diabetes have widened, narrowed, or remained stable over the past decade and to what extent they contribute to broader health inequalities. Understanding these patterns can help to identify vulnerable populations and inform targeted strategies to improve diabetes prevention and care, particularly in socially disadvantaged regions.

Therefore, we aimed to estimate trends in the prevalence of diagnosed type 2 diabetes by regional deprivation in Germany from 2011 to 2023.

## Methods

### Data source

In this ecological study, we used nationwide claims data from all German statutory health (GKV, gesetzliche Krankenversicherung) aggregated on county level, provided by the Central Institute for Ambulatory Health Care in Germany (Zi), to estimate the age- and sex-specific prevalence of type 2 diabetes by regional deprivation for the years 2011 to 2023. These data comprised all German statutory insured individuals, approximately 85% of the total population in Germany, with at least one outpatient visit per year on the level of Germany’s 401 districts (‚Landkreise und kreisfreie Städte‘). We excluded cases with missing residence data and age ≥ 111 years. The Zi ensured anonymity of individuals by aggregating the data by age (5-year age groups (< 50 to ≥ 85 years) and sex (men, women) with a minimum of 30 cases per stratum. We linked publicly available data on the GISD to the individuals’ district of residence [8]. The GISD is based on the “Indicators and Maps of Spatial and Urban Development” (INKAR) database provided by the Federal Institute for Research on Building, Urban Affairs and Spatial Development (as of 31 December 2019) [8, 9]. It comprises nine indicators across three dimensions, i.e., education, occupation, and income, and is recalculated annually to reflect changes in regional socioeconomic conditions. All 401 districts in Germany are classified into quintiles of socioeconomic deprivation: ‘very low’ (GISD quintile 1) to ‘very high’ (GISD quintile 5). Since the GISD quintiles are defined based on the distribution of districts rather than population size, each quintile contains an equal number of districts, but the number of inhabitants may vary across quintiles.

### Definition of type 2 diabetes

As in previous studies [3, 10] we identified prevalent cases of type 2 diabetes based on the 10th revision of the International Classification of Diseases (ICD-10): E11 (type 2 diabetes), E12 (malnutrition-related diabetes mellitus), E13 (other specified diabetes mellitus), and E14 (unspecified diabetes mellitus), or combinations of these codes. To minimize the risk of false positives, we considered only confirmed diagnoses, present with two diagnoses in two different quarters in a given year (M2Q criterion). Further details can be found elsewhere [10].

### Statistical methods

We estimated the crude and age-standardised prevalence of type 2 diabetes, using the study population from 2011 as the reference. Additionally, we estimated the prevalence by strata of sex, age and quintiles of the GISD.

To assess the association between socioeconomic deprivation and prevalence of type 2 diabetes, we used log-binomial regression to estimate prevalence ratios. We included age, sex and GISD quintiles as categorical variables and a natural cubic spline with two knots for calendar year. To examine changes in GISD-related inequalities in type 2 diabetes prevalence over time across sex and age groups, we included a four-way interaction (and all lower-level interactions, three degrees of freedom) between age, sex, GISD and calendar year. We used cluster-robust standard errors to account for the correlation of observations in 401 counties.

We reported following the STROBE guideline [11]. All analyses were performed using R version 4.4.0 statistical software (R Foundation for Statistical Computing) and SAS 9.4 SAS/STAT 15.3 (SAS Institute Inc. Cary, NC, USA).

## Results

### Study population

**Fig. 1:**
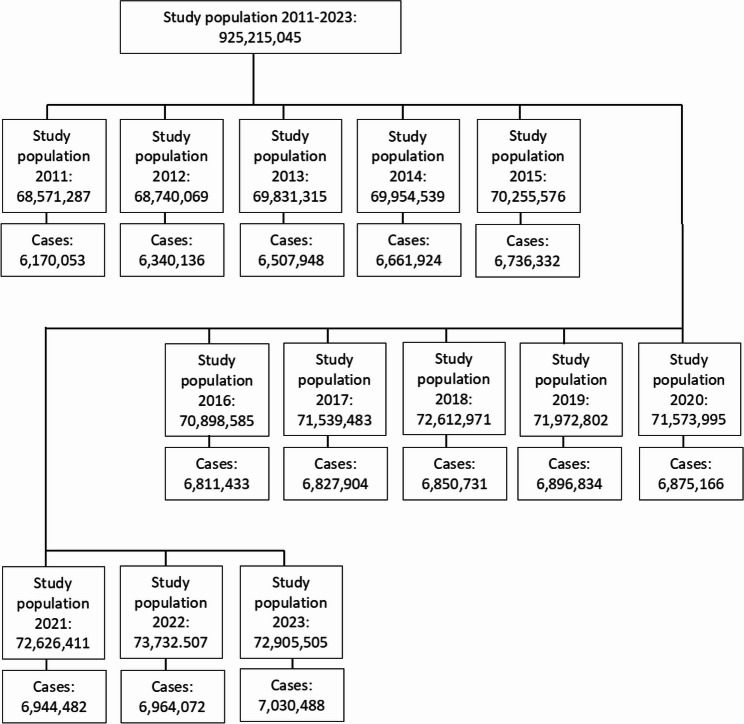
Flow chart illustrating study population formation and annual derivation, 2011–2023, Germany

In total, we included 68.6 to 73.7 million persons under risk in each reporting year (Fig. 1, Additional file 1). There were more women than men (54.3% vs. 45.7%). Regarding GISD, the number of persons showed a declining trend (besides GISD 2 to GISD 3) with increasing deprivation (e.g., 2023 GISD quintile 1: 17.3 million, 23.7%; GISD quintile 5: 11.0 million, 15.1%) (Additional Table S1). In the following sections, we describe results for crude age- and sex-specific prevalence as well as age-standardised prevalence.

### Crude prevalence of type 2 diabetes

**Fig. 2:**
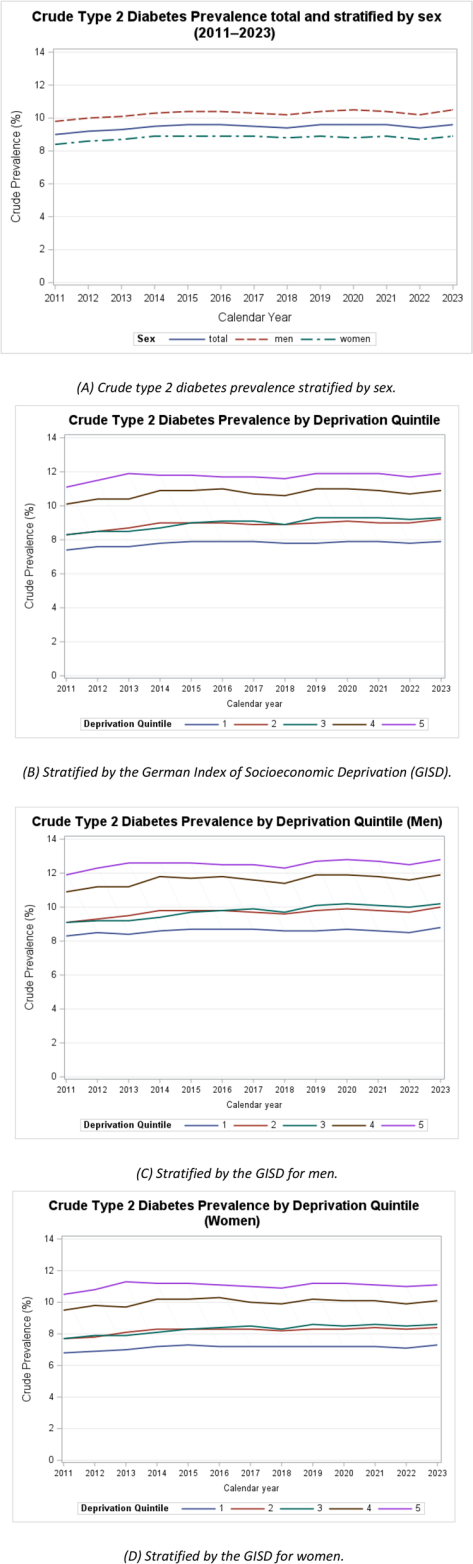
Crude type 2 diabetes prevalence 2011–2023, Germany. **A** Crude type 2 diabetes prevalence stratified by sex. **B** Stratified by the German Index of Socioeconomic Deprivation, (GISD). **C** Stratified by the GISD for men. **D** Stratified by the GISD for women. Confidence intervals are not displayed due to the high precision. GISD from 1 (very low) to 5 (very high)

The overall crude prevalence of type 2 diabetes increased from 9.00% (95% CI: 8.99–9.01) in 2011 to 9.60% (9.59–9.61) in 2023 (Fig. 2A and Additional Table S2). When stratified by sex, men consistently exhibited higher prevalence than women. Among men, the crude prevalence increased from 9.80% (9.79–9.81) in 2011 to 10.60% (10.59–10.61) in 2023, whereas in women, it increased less than in men, from 8.40% (8.39–8.41) in 2011 to 8.80% (8.79–8.81) in 2023.

We observed a clear socioeconomic gradient, with a higher crude prevalence of type 2 diabetes in more deprived regions (Fig. 2B and Additional Table S2). In 2011, the crude prevalence ranged from 7.40% (7.39–7.41) in the least deprived districts (GISD 1) to 11.20% (11.18–11.22) in the most deprived districts (GISD 5). By 2023, this pattern persisted, with prevalence of 8.00% (7.99–8.01) in GISD 1 and 11.80% (11.78–11.82) in GISD 5.

When further stratified by sex, men had consistently higher crude prevalence than woman across all levels of deprivation. In 2011, the crude prevalence of type 2 diabetes in men ranged from 8.20% (8.18–8.22) in the least deprived districts (GISD 1) to 11.99% (11.98–12.03) in the most deprived districts (GISD 5), increasing to 8.80% (8.78–8.82; GISD 1) and 12.80% (12.77–12.83; GISD 5) in 2023, respectively (Fig. 2C and Table S2). Among women, prevalence decreased slightly from 7.40% (7.39–7.41) in the least deprived districts (GISD 1) in 2011 to 7.20% (7.18–7.22; GISD 1) in 2023 and increased slightly in the most deprived districts (GISD 5) from 10.40% (10.38–10.42; GISD 1) in 2011 to 11.00% (10.98–11.03; GISD 5) in 2023 (Fig. 2D and Additional file 2).

**Fig. 3:**
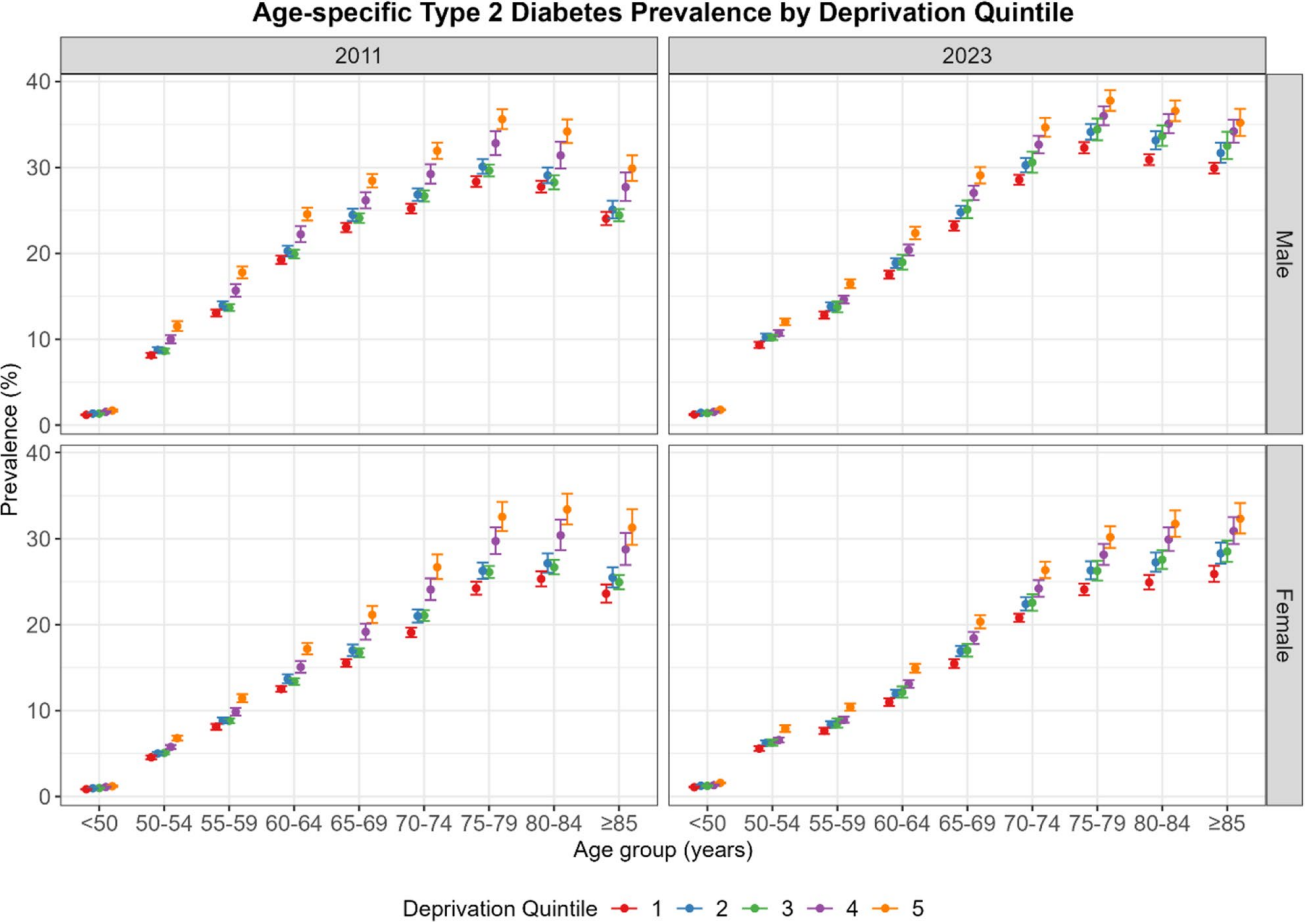
Predicted type 2 diabetes prevalence by age, sex and deprivation quintile, 2011 and 2023, Germany. Estimates based on log-binomial regression model. GISD from 1 (very low) to 5 (very high)

When further stratified by age, we observed GISD-related inequalities in all age groups in 2011 and in 2023 (Fig. 3). Type 2 diabetes prevalence generally increased with age, reaching a peak in the 75–84 age group before declining in older ages. An exception was observed among women in 2023, where prevalence continued to rise even in the oldest age group. Overall prevalence was higher in more deprived regions, although in 2011, prevalence in GISD quintile 2 was slightly higher than in quintile 3 across all age groups.

### Age-standardised prevalence of type 2 diabetes

**Fig. 4:**
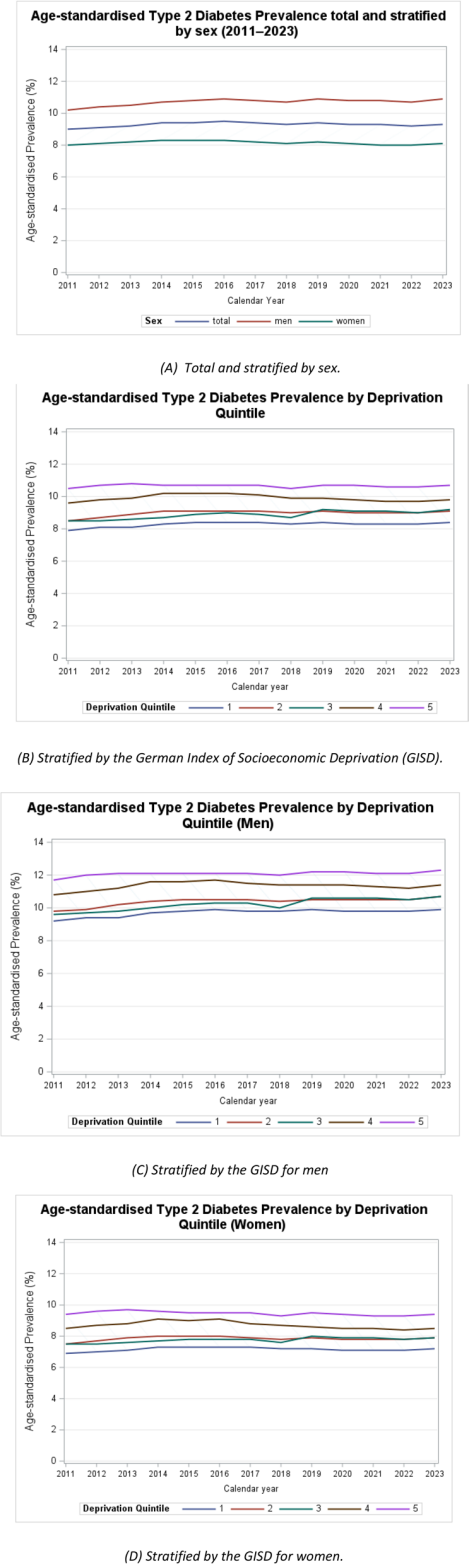
Age-standardised type 2 diabetes prevalence 2011–2023, Germany. **A** Total and stratified by sex. **B** Stratified by the German Index of Socioeconomic Deprivation, (GISD), (**C**) Stratified by the GISD for men, **D** Stratified by the GISD for women. Confidence intervals are not displayed due to the high precision. GISD from 1 (very low) to 5 (very high)

The age-standardised prevalence of type 2 diabetes followed a similar trend, but the trend was less pronounced than in the crude prevalence. The overall age-standardised prevalence slightly increased from 9.00% (8.99-9.00) in 2011 to 9.35% (9.34–9.35) in 2023 (Fig. 4A and Additional Table S2). Age-standardisation increased the prevalence in men while lowering it in women. Among men, the age-standardised prevalence increased from 10.24% (10.23–10.25) in 2011 to 10.93% (10.92–10.94) in 2023. In contrast, the prevalence in women remained relatively stable, ranging from 7.98% (7.97–7.99) in 2011 to 8.08% (8.07–8.08) in 2023.

A socioeconomic gradient was also evident in the age-standardised prevalence, though it was less pronounced compared to the crude rates (Fig. 4B and Additional Table S2). In the least deprived districts (GISD 1), the age-standardised prevalence slightly increased from 7.91% (7.90–7.93) in 2011 to 8.38% (8.37–8.39) in 2023, while in the most deprived districts (GISD 5), it remained relatively stable, with 10.46% (10.45–10.48) in 2011 and 10.69% (10.67–10.71) in 2023.

When further stratified by sex, age-standardisation led to a slight increase in prevalence among men in the least deprived districts (GISD 1, 9.19%, 9.17–9.21 in 2011 to 9.94%, 9.92–9.96 in 2023) and in the most deprived districts (GISD 5, 11.73%, 11.71–11.75 in 2011 to 12.29%, 12.27–12.32 in 2023) (Fig. 4C and Additional Table S2). In women, the age-standardized rates remained stable, with 6.92% (6.90–6.93) in the least deprived districts (GISD 1) and 9.39% (9.38–9.41) in the most deprived districts in 2011, compared to 7.17% (7.16–7.19) and 9.37% (9.35–9.39) in 2023 (Fig. 4D and Table S2).

### Prevalence ratios: socioeconomic gradient in type 2 diabetes prevalence

The relative differences in prevalence of type 2 diabetes by socioeconomic deprivation were more pronounced in women than in men (Table 1). In 2011, the prevalence of type 2 diabetes was 34% higher in women (prevalence ratio (PR): 1.34, 1.27–1.41) and 25% higher in men (PR: 1.25, 1.20–1.30) in the most deprived districts (GISD 5) compared to the least deprived districts (GISD 1). This socioeconomic gradient persisted in 2023, with PRs of 1.29 (1.23–1.35) in women and 1.22 (1.17–1.26) in men. In 2023, type 2 diabetes prevalence was 10% higher in women (PR: 1.10, 1.05–1.15) and 8% higher in men (PR: 1.08, 1.03–1.13) in the medium deprived districts (GISD 3) compared to the least deprived districts (GISD 1).

**Table 1:** Prevalence ratios and 95% confidence intervals for type 2 diabetes by sex and deprivation

Regional socioeconomic deprivation	2011				2023			
	Women		Men		Women		Men	
	PR	95% CI	PR	95% CI	PR	95% CI	PR	95% CI
GISD 2 vs. 1	1.09	(1.04–1.14)	1.06	(1.02–1.10)	1.09	(1.05–1.14)	1.07	(1.03–1.10)
GISD 3 vs. 1	1.06	(1.01–1.10)	1.02	(0.99–1.06)	1.10	(1.05–1.15)	1.08	(1.03–1.13)
GISD 4 vs. 1	1.25	(1.18–1.31)	1.18	(1.13–1.24)	1.18	(1.13–1.24)	1.14	(1.10–1.18)
GISD 5 vs. 1	1.34	(1.27–1.41)	1.25	(1.20–1.30)	1.29	(1.23–1.35)	1.22	(1.17–1.26)

2011 & 2023, Germany. *GISD* German Index of Socioeconomic Deprivation (1 = very low, 2–4 = medium, 3 = very high), *CI* Confidence interval, *PR* Prevalence ratio. *n* = 423,108,001 women, *n* = 502,107,044 men

## Discussion

Based on data of about 70 million statutorily insured people, we show that the crude and age-standardised prevalence of type 2 diabetes increased only slightly between 2011 and 2023, with a consistently higher prevalence in men. A clear socioeconomic gradient emerged: individuals in more deprived regions had higher prevalence, particularly among women. While crude prevalence slightly increased across all deprivation levels, age-standardised trends were less pronounced, indicating that much of the observed increase is attributable to population aging rather than a substantial increase in underlying diabetes risk. GISD-related inequalities persisted over time, but trends varied by age, and overall growth in prevalence remained relatively slow.

Our findings are partially consistent with national and international research. The Global Burden of Diseases Study reported an increasing type 2 diabetes prevalence among individuals aged 15–34 years from 1990 to 2021, particularly in regions with a low to middle Socio-Demographic Index (SDI). This shows, although type 2 diabetes is considered a disease of older adults, early onset type 2 diabetes is increasingly recognized. As we focused on adults aged 18–85 + years, these findings are particularly relevant for the lower end of our age spectrum, as they indicate that the rise in type 2 diabetes prevalence is not confined to older adults but also affects younger adults within our population, highlighting the importance of early prevention and intervention strategies. Furthermore, low SDI was associated not only with a higher risk of developing type 2 diabetes, but also with more severe disease progression and complications [12]. Moreover, type 2 diabetes has been identified as a key driver of mortality and disability-adjusted life years, particularly in low and low-middle SDI regions, highlighting the disproportionate burden of disease in socioeconomically disadvantaged populations [13]. However, higher SDI regions had consistently higher prevalence and growing disparities [13]. In the US, age-standardised type 2 diabetes prevalence rose from 10.2% in 2012 to 12.2% in 2022 [14] and several studies found strong associations between socioeconomic deprivation and diabetes risk [15, 16]. In England between 2018 and 2023, up to 64% of diabetes cases were attributable to socioeconomic deprivation [17]. The British Women’s Heart and Health Study estimated an odds ratio (OR) of 1.32 (1.13–1.53) per quintile increase in deprivation [18].

In Germany, previous studies estimated type 2 diabetes prevalence at 8.6% in the most deprived regions in 2009–2010 [5], and 7.0% vs. 11.3% (least vs. most deprived) between 2013 and 2017 [19]. Older studies also showed increases in type 2 diabetes risk by increasing deprivation level after adjusting for sex, age and lifestyle factors [6, 20]. For example, ORs of 1.88 (1.16–2.04; [20]) and 1.36 (1.13–1.63; [6]) were estimated for GISD quintile 4, while for GISD quintile 5, ORs of 2.14 (1.23–3.55; [20]) and 1.66 (1.37-2.00; [6]) were observed compared to quintile 1. Similar patterns in type 2 diabetes incidence have been reported in Finland [21], Scotland [22], or Sweden [23]. In Germany, type 2 diabetes incidence was more than twice as high in the most deprived regions compared to the least deprived areas [24]. Another study also reported on sex-specific differences, with stronger associations between deprivation and type 2 diabetes among women [5].

While we used the GISD to measure deprivation, other studies [5, 6, 20] relied on the German Index of Multiple Deprivation (GIMD), which takes a broader approach by incorporating seven dimensions: income, employment, education, community, environment, safety, and health [7]. In international research the SDI is commonly used as composite measure based on income per capita, average educational attainment, and fertility rates [25]. Despite the differences in the indices used, the consistent results across studies and countries reinforce the robustness of the association between regional deprivation and type 2 diabetes. Key factors such as income, education, and employment emerge as primary drivers of the observed disparities, highlighting the critical role social determinants play in the development of type 2 diabetes. Since the GISD is recalculated annually, changes in regional socioeconomic conditions may shift districts between deprivation quintiles over time. For example, a district with high diabetes prevalence that improves socioeconomically may move to a lower deprivation quintile, potentially attenuating observed disparities even if the underlying health burden remains unchanged. Conversely, districts with worsening deprivation may appear to amplify inequalities. Such shifts highlight that temporal trends reflect not only the persistence or widening of health disparities, but also structural socioeconomic developments at the regional level.

Our study period includes the COVID-19 pandemic (2020–2021), which disrupted routine healthcare access and diagnostic activities for many individuals [26]. These changes could have temporarily affected the detection and documentation of type 2 diabetes, potentially leading to short-term fluctuations in type 2 diabetes prevalence. In addition, pandemic-related shifts in diet, physical activity, and stress may have influenced diabetes risk [27], particularly among socioeconomically vulnerable groups. While these factors likely had a limited impact on long-term trends, they should be considered when interpreting prevalence patterns during this period.

### Strengths and limitations

Our study has two main strengths. First, our estimates are based on a large data set covering 70 million statutorily insured people, providing robust prevalence estimates. Second, we cover a long observation period of 13 years, allowing for an analysis of temporal trends in type 2 diabetes prevalence and deprivation-related inequalities.

However, several limitations should be considered when interpreting the results. Our case definition, namely an individual with at least two confirmed type 2 diabetes diagnoses within the same calendar year, may have led to differential misclassification by socioeconomic position. Individuals in more deprived areas may be more likely to have undiagnosed or undocumented diabetes [28], which could result in an underestimation of prevalence in this groups, and, as a consequence, the socioeconomic disparities observed in our study may be underestimated. Although the definition we used to define type 2 diabetes with this data source is well established, there is unfortunately no formal validation study providing objective validity measures (e.g., sensitivity and specificity).

Furthermore, our analysis is restricted to individuals with statutorily health insurance, who account for approximately 85% of the German population. The remaining 15% of the population are primarily privately insured or, in rare cases, uninsured. Privately insured individuals are generally of higher socioeconomic position, tend to be healthier, have greater access to and utilization of healthcare services than those with statutory insurance [29, 30], and have a lower prevalence of type 2 diabetes [4]. Therefore, while our findings accurately reflect the majority of the German population, extrapolation to the entire population may slightly overestimate prevalence, particularly if the healthier privately insured segment is underrepresented.

In addition, individuals without any form of health insurance are not captured in our data. The extent to which diabetes is present in this group remains unknown, potentially introducing further bias. Moreover, the introduction of structured Disease Management Programs for diabetes, which provide financial or administrative incentives for enrolling patients, may have led to changes in documentation practices and diagnostic coding over time. Finally, changes in the proportion of undiagnosed type 2 diabetes cases – potentially varying by sex or level of deprivation, may have influenced the observed temporal trends and patterns of social inequality.

## Conclusion

Our findings indicate persistent socioeconomic disparities in the prevalence of type 2 diabetes over time, with a consistent association between higher deprivation and increased diabetes prevalence. The socioeconomic gradient is more pronounced among women, suggesting a potential sex-specific vulnerability to the influence of socioeconomic factors on diabetes risk. While these analyses demonstrate clear associations at the population level, causal inferences cannot be drawn from ecological data, and individual-level determinants were not assessed, which introduces the potential for ecological fallacy. Future studies should explore the mechanisms behind socioeconomic disparities in type 2 diabetes, focusing on sex differences and key social determinants and evaluate targeted interventions in high-risk populations.

## Supplementary Information

Supplementary Material 1.

Supplementary Material 2.

## Data Availability

The data that support the findings of this study are available from the Central Institute for Ambulatory Health Care in Germany (Zi), but restrictions apply to the availability of these data, which were used under license for the current study, and so are not publicly available. Data are however available from the authors upon reasonable request and with permission of Zi.

## Abbreviations

CI: Confidence interval
GISD: German Index of Socioeconomic Deprivation
PR: Prevalence Ratio
SDI: Socio-Demographic Index
