# Supplementary material for: Prevalence of type 2 diabetes from 2011 to 2023 by regional socioeconomic deprivation in Germany: an ecological study
Source: BMC Public Health. 2025 Dec 17;26:258. doi: 10.1186/s12889-025-25908-x (PMC12821838; doi:10.1186/s12889-025-25908-x)
Supplement: Supplementary file 1 — Supplementary Material 1. [file 12889_2025_25908_MOESM1_ESM.docx]

Table S1.

Study population characteristics stratified by deprivation quintile, 2011-2023, Germany

| **Characteristics** | **GISD 1** | **GISD 2** | **GISD 3** | **GISD 4** | **GISD 5** | **Total** |
| --- | --- | --- | --- | --- | --- | --- |
|  | N (%) | N (%) | N (%) | N (%) | N (%) | N (%) |
| **Total** | 217,797,428 (23.5%) | 187,604,713 (20.3%) | 189,101,884 (20.4%) | 177,778,972 (19.2%) | 152,932,048 (16.5%) | 925,215,045 (100%) |
| **Sex** | | | | | | |
| *Men* | 98,545,151 (23.3%) | 85,814,697 (20.3%) | 87,115,876 (20.6%) | 81,345,429 (19.2%) | 70,286,848 (16.6%) | 423,108,001 (45.7%) |
| *Women* | 119,252,277 (23.8%) | 101,790,016 (20.3%) | 101,986,008 (20.3%) | 96,433,543 (19.2%) | 82,645,200 (16.5%) | 502,107,044 (54.3%) |
| **Age [in years]** | | | | | | |
| *<50* | 128,424,555 (25.0%) | 106,112,206 (20.7%) | 105,105,851 (20.5%) | 94,291,595 (18.4%) | 79,680,918 (15.5%) | 513,615,125 (55.5%) |
| *50-54* | 16,277,407 (23.0%) | 14,414,051 (20.4%) | 14,731,058 (20.8%) | 13,552,825 (19.2%) | 11,747,402 (16.6%) | 70,722,743 (7.6%) |
| *55-59* | 15,154,508 (22.0%) | 13,836,924 (20.1%) | 14,395,311 (20.9%) | 13,554,494 (19.7%) | 11,938,716 (17.3%) | 68,879,953 (7.4%) |
| *60-64* | 12,816,477 (21.2%) | 11,917,803 (19.7%) | 12,441,550 (20.6%) | 12,320,844 (20.4%) | 10,970,766 (18.1%) | 60,467,440 (6.5%) |
| *65-69* | 10,711,332 (21.2%) | 9,864,568 (19.6%) | 10,192,321 (20.2%) | 10,485,143 (20.8%) | 9,202,266 (18.2%) | 50,455,630 (5.5%) |
| *70-74* | 10,263,841 (21.6%) | 9,244,582 (19.4%) | 9,370,587 (19.7%) | 9,866,828 (20.7%) | 8,867,576 (18.6%) | 47,613,414 (5.1%) |
| *75-79* | 9,577,983 (21.4%) | 8,701,049 (19.4%) | 8,869,419 (19.8%) | 9,431,431 (21.0%) | 8,273,302 (18.4%) | 44,853,184 (4.8%) |
| *80-84* | 7,599,169 (21.0%) | 7,087,757 (19.6%) | 7,360,225 (20.4%) | 7,542,352 (20.9%) | 6,559,884 (18.1%) | 36,149,387 (3.9%) |
| *≥85* | 6,972,156 (21.5%) | 6,425,773 (19.8%) | 6,635,562 (20.4%) | 6,733,460 (20.7%) | 5,691,218 (17.5%) | 32,458,169 (3.5%) |
| **Year** | | | | | | |
| *2011* | 16,213,668 (23.6%) | 13,926,443 (20.3%) | 12,797,527 (18.7%) | 10,921,447 (15.9%) | 14,712,202 (21.5%) | 68,571,287 (7.4%) |
| *2012* | 16,171,175 (23.5%) | 13,579,445 (19.8%) | 13,497,984 (19.6%) | 11,307,664 (16.4%) | 14,183,801 (20.6%) | 68,740,069 (7.4%) |
| *2013* | 16,266,100 (23.3%) | 13,494,321 (19.3%) | 13,516,919 (19.4%) | 14,399,310 (20.6%) | 12,154,665 (17.4%) | 69,831,315 (7.5%) |
| *2014* | 16,390,309 (23.4%) | 13,611,397 (19.5%) | 13,713,386 (19.6%) | 14,860,866 (21.2%) | 11,378,581 (16.3%) | 69,954,539 (7.6%) |
| *2015* | 16,537,743 (23.5%) | 14,011,221 (19.9%) | 13,353,506 (19.0%) | 15,029,799 (21.4%) | 11,323,307 (16.1%) | 70,255,576 (7.6%) |
| *2016* | 16,874,979 (23.8%) | 14,175,431 (20.0%) | 13,343,658 (18.8%) | 14,970,577 (21.1%) | 11,533,940 (16.3%) | 70,898,585 (7.7%) |
| *2017* | 16,854,218 (23.6%) | 14,614,512 (20.4%) | 13,047,479 (18.2%) | 15,738,507 (22.0%) | 11,284,767 (15.8%) | 71,539,483 (7.7%) |
| *2018* | 16,747,221 (23.1%) | 14,825,953 (20.4%) | 13,929,637 (19.2%) | 15,579,182 (21.5%) | 11,530,978 (15.9%) | 72,612,971 (7.8%) |
| *2019* | 16,925,682 (23.5%) | 14,920,926 (20.7%) | 16,238,789 (22.6%) | 12,942,779 (18.0%) | 10,944,626 (15.2%) | 71,972,802 (7.8%) |
| *2020* | 16,898,775 (23.6%) | 14,854,665 (20.8%) | 16,148,778 (22.6%) | 12,835,040 (17.9%) | 10,836,737 (15.1%) | 71,573,995 (7.7%) |
| *2021* | 17,183,172 (23.7%) | 15,104,153 (20.8%) | 16,390,071 (22.6%) | 12,990,694 (17.9%) | 10,958,321 (15.1%) | 72,626,411 (7.8%) |
| *2022* | 17,456,683 (23.7%) | 15,332,491 (20.8%) | 16,644,023 (22.6%) | 13,181,666 (17.9%) | 11,117,644 (15.1%) | 73,732,507 (8.0%) |
| *2023* | 17,277,703 (23.7%) | 15,153,755 (20.8%) | 16,480,127 (22.6%) | 13,021,441 (17.9%) | 10,972,479 (15.1%) | 72,905,505 (7.9%) |

Notes. GISD, German Index of Socioeconomic Deprivation (1=very low, 2-4=medium, 3=very high). The values in the columns GISD 1 to GISD 5 represent row percentages; the values in the ‘Total’ column represent column percentages.
