# Supplementary material for: Prevalence of type 2 diabetes from 2011 to 2023 by regional socioeconomic deprivation in Germany: an ecological study
Source: BMC Public Health. 2025 Dec 17;26:258. doi: 10.1186/s12889-025-25908-x (PMC12821838; doi:10.1186/s12889-025-25908-x)
Supplement: Supplementary file 2 — Supplementary Material 2. [file 12889_2025_25908_MOESM2_ESM.docx]

Table S2.

Crude and age-standardised type 2 diabetes prevalence by sex and deprivation quintile 1 and 5.

| **Year** | **Men** | | | | **Women** | | | | **Total** | | | |
| --- | --- | --- | --- | --- | --- | --- | --- | --- | --- | --- | --- | --- |
|  | *Crude prevalence [%]* | *95% CI* | *Age-std. prevalence [%]* | *95% CI* | *Crude prevalence [%]* | *95% CI* | *Age-std. prevalence [%]* | *95% CI* | *Crude prevalence [%]* | *95% CI* | *Age-std. prevalence [%]* | *95% CI* |
| *2011* | 9.80 | (9.79-9.81) | 10.24 | (10.23-10.25) | 8.40 | (8.39-8.41) | 7.98 | (7.97-7.99) | 9.00 | (8.99-9.01) | 9.00 | (8.99-9.00) |
| *GISD 1* | 8.20 | (8.18-8.22) | 9.19 | (9.17-9.21) | 7.40 | (7.39-7.41) | 6.92 | (6.90-6.93) | 7.40 | (7.39-7.41) | 7.91 | (7.90-7.93) |
| *GISD 5* | 11.99 | (11.98-12.03) | 11.73 | (11.71-11.75) | 10.40 | (10.38-10.42) | 9.39 | (9.38-9.41) | 11.20 | (11.18-11.22) | 10.46 | (10.45-10.48) |
| *2023* | 10.60 | (10.59-10.61) | 10.93 | (10.92-10.94) | 8.80 | (8.79-8.81) | 8.08 | (8.07-8.08) | 9.60 | (9.59-9.61) | 9.35 | (9.34-9.35) |
| *GISD 1* | 8.80 | (8.78-8.82) | 9.94 | (9.92-9.96) | 7.20 | (7.18-7.22) | 7.17 | (7.16-7.19) | 8.00 | (7.99-8.01) | 8.38 | (8.37-8.39) |
| *GISD 5* | 12.80 | (12.77-12.83) | 12.29 | (12.27-12.32) | 11.00 | (10.98-11.03) | 9.37 | (9.35-9.39) | 11.80 | (11.78-11.82) | 10.69 | (10.67-10.71) |

*Notes*. 2011 & 2023, Germany. GISD, German Index of Socioeconomic Deprivation (1=very low, 2-4=medium, 3=very high); CI, confidence interval; std, standardised.
